# Analysing governments' progress on the right to health

**DOI:** 10.2471/BLT.23.290184

**Published:** 2024-03-14

**Authors:** Alicia Ely Yamin, Luciano Bottini Filho, Camila Gianella Malca

**Affiliations:** a Harvard University, Harvard Law School, 23 Everett Street, Cambridge 02138 MA, United States of America.; b Helena Kennedy Centre for International Justice, Sheffield Hallam University, Sheffield, England.; c Department of Social Sciences, Pontificia Universidad Católica del Perú, Lima, Peru.

## Abstract

**Objective:**

To examine the influence of varying articulations of the right to health under domestic constitutions, legislation and jurisprudence on the scope of legal protection for health.

**Methods:**

We investigated legal recognition of the right to health, by conducting a three-level search. First, we searched databases containing constitutional texts. Second, we did a thematic analysis of those constitutional texts with explicit constitutional recognition of health rights, employing NVivo for coding. For the 54 World Health Organization (WHO) Member States without explicit constitutional provisions, we explored statutory paths, judicial constructions and instances where both methods contributed to the acknowledgement of health rights. Lastly, we confirmed evidence of jurisprudence constructing a right to health based on a combination of domestic law and international human rights norms incorporated directly into the text.

**Findings:**

We identified 140 WHO Member States with a constitutionalized right to health. Our analysis suggests there are notable variations in the legal scope of protection for health, including breadth of entitlements and the possibility of enforcing these rights through the legal system. We also highlight the critical importance of constitutional acknowledgement, legislative measures, and judicial interpretations in shaping the legal entitlements to health-care services, affecting their accessibility and financial support.

**Conclusion:**

The analysis offers insights for policy-makers to assess different approaches to health-related entitlements, with implications for health financing and the evaluation of Member States' strides towards universal access to comprehensive care. This analysis also illuminates how distinct formulations of the right to health have varied effects on reducing health disparities.

## Résumé

**Objectif:**

Étudier l’impact de diverses formulations du droit à la santé selon les constitutions, la législation et la jurisprudence nationales sur le champ d'application de la protection juridique en matière de santé.

**Méthodes:**

Nous avons examiné la reconnaissance juridique du droit à la santé en effectuant une recherche à trois niveaux. Tout d'abord, nous avons exploré des bases de données contenant des textes constitutionnels. Ensuite, nous avons procédé à une analyse thématique de ces textes constitutionnels reconnaissant explicitement les droits à la santé, en utilisant NVivo pour le codage. Pour les 54 États Membres de l'Organisation mondiale de la Santé (OMS) dépourvus de dispositions constitutionnelles, nous avons passé en revue les parcours légaux, instances et interprétations judiciaires où les deux méthodes ont contribué à la reconnaissance de droits à la santé. Enfin, nous avons corroboré les preuves de jurisprudence formulant un droit à la santé fondé sur la combinaison, directement intégrée dans le texte, d’une législation nationale et de normes internationales en matière de droits humains.

**Résultats:**

Nous avons identifié 140 États Membres de l'OMS ayant constitutionnalisé le droit à la santé. Notre analyse suggère qu'il existe des variations considérables dans le champ d'application de la protection juridique en matière de santé, notamment dans la portée de ces droits et la possibilité de les faire respecter par la voie légale. Nous soulignons en outre l'importance cruciale de la reconnaissance constitutionnelle, des mesures législatives et des interprétations judiciaires dans la formulation des droits juridiques aux services de soins de santé, du fait de son impact sur leur accessibilité et sur le soutien financier.

**Conclusion:**

La présente analyse fournit des informations aux responsables politiques afin qu'ils puissent examiner plusieurs approches relatives aux droits de la santé, avec des implications en termes de financement de la santé et l'évaluation des progrès réalisés par les États Membres dans le cadre de l'accès universel à des soins complets. Elle met également en lumière la diversité des effets qu'exercent les différentes formulations du droit à la santé sur la réduction des inégalités en la matière.

## Resumen

**Objetivo:**

Examinar la influencia de las diferentes articulaciones del derecho sobre la salud en las constituciones, la legislación y la jurisprudencia nacionales sobre el alcance de la protección jurídica de la salud.

**Métodos:**

Se investigó el reconocimiento jurídico del derecho sobre la salud mediante una búsqueda en tres niveles. En primer lugar, se buscaron bases de datos que incluyeran textos constitucionales. En segundo lugar, se realizó un análisis temático de los textos constitucionales con reconocimiento constitucional explícito de los derechos sobre la salud, utilizando NVivo para la codificación. En el caso de los 54 Estados Miembros de la Organización Mundial de la Salud (OMS) sin disposiciones constitucionales explícitas, se exploraron las vías estatutarias, las construcciones judiciales y los casos en que ambos métodos contribuyeron al reconocimiento de los derechos sobre la salud. Por último, se confirmaron los casos en los que la jurisprudencia había establecido un derecho sobre la salud basado en una combinación de legislación nacional y normas internacionales de derechos humanos incorporadas directamente al texto.

**Resultados:**

Se identificaron 140 Estados Miembros de la OMS con un derecho sobre la salud constitucionalizado. El análisis sugiere que existen notables variaciones en el alcance jurídico de la protección de la salud, incluida la extensión de los derechos y la posibilidad de hacerlos valer a través del sistema jurídico. También se destaca la importancia decisiva del reconocimiento constitucional, las medidas legislativas y las interpretaciones judiciales en la configuración de los derechos legales a los servicios de atención sanitaria, que afectan a su accesibilidad y apoyo financiero.

**Conclusión:**

El análisis ofrece información para que los responsables de formular las políticas evalúen los diferentes enfoques de los derechos relacionados con la salud, con implicaciones para la financiación sanitaria y la evaluación de los avances de los Estados Miembros hacia el acceso universal a una atención integral. Este análisis también aclara cómo las diversas formulaciones del derecho sobre la salud tienen efectos variados en la reducción de las disparidades sanitarias.

## ملخص

**الغرض:**

دراسة تأثير الصيغ المتباينة للتعبير عن الحق في التمتع بالصحة بموجب الدساتير، والتشريعات، والنظم القانونية المحلية، بخصوص نطاق الحماية القانونية للصحة.

**الطريقة:**

قمنا بالتحقيق في الاعتراف القانوني بالحق في التمتع بالصحة، من خلال إجراء بحث على ثلاثة مستويات. أولاً، قمنا بالبحث في قواعد البيانات التي تحتوي على النصوص الدستورية. ثانيًا، قمنا بإجراء تحليل موضوعي لتلك النصوص الدستورية التي تتضمن اعترافًا دستوريًا صريحًا بالحقوق الصحية، مع استخدام نظام NVivo في الترميز. بالنسبة للدول الـ 54 الأعضاء في منظمة الصحة العالمية (WHO)، والتي ليس لديها أحكام دستورية صريحة، قمنا باستكشاف المسارات القانونية، والهياكل القضائية، والحالات التي ساهمت فيها كلتا الطريقتين في الاعتراف بالحقوق الصحية. وأخيرًا، قمنا بتأكيد الأدلة على أن النظام القانوني يؤسس الحق في التمتع بالصحة على أساس الدمج بين القانون المحلي والمعايير الدولية لحقوق الإنسان المضمنة مباشرة في النص.

**النتائج:**

توصلنا لتحديد وجود 140 دولة من الأعضاء في منظمة الصحة العالمية تمنح الحق الدستوري في التمتع بالصحة. ويشير تحليلنا إلى وجود تباينات ملحوظة في النطاق القانوني لحماية الصحة، بما في ذلك نطاق الاستحقاقات وإمكانية تطبيق هذه الحقوق من خلال النظام القانوني. كما نسلط الضوء أيضًا على الأهمية الحيوية للاعتراف الدستوري، والتدابير التشريعية، والتفسيرات القضائية في تشكيل الاستحقاقات القانونية لخدمات الرعاية الصحية، مما يؤثر على إمكانية الحصول عليها والدعم المالي.

**الاستنتاج:**

يقدم التحليل لواضعي السياسات النظرة المتعمقة لتقييم الأساليب المختلفة للاستحقاقات المتعلقة بالصحة، مع الآثار المترتبة على تمويل الصحة، وتقييم خطوات الدول الأعضاء نحو تسهيل حصول العامة على الرعاية الشاملة. كما يُلقي هذا التحليل أيضًا الضوء على مدى تأثير الصيغ المتباينة للتعبير عن الحق في التمتع بالصحة، على الحد من التفاوتات الصحية.

## 摘要

**目的:**

研究国内宪法、法规和法律体系项下不同的健康权规定对法定健康保护范围的影响。

**方法:**

通过开展三级研究，我们探讨了健康权的法律认可情况。首先，我们检索了包含宪法文本的数据库。其次，我们对那些有明确立法认可健康权的宪法文本进行了主题分析，并使用 Nvivo 对其进行了编码。对于没有明确宪法规定的 54 个世界卫生组织 (WHO) 成员国，我们探讨了有助于认可健康权的法定途径、司法解释以及两种方法均适用的实例。最后，在结合国内法和直接纳入立法文本的国际人权规范的基础上，我们确认了构建健康权的法定证据。

**结果:**

我们发现，有 140 个世界卫生组织成员国将健康权纳入了宪法范围。我们的分析表明，各国在健康保护相关法律范围方面存在显著差异，包括应享有权利的范围以及依法行使这些权利的可能性。我们还强调，宪法认可、立法措施和司法解释在确保卫生保健服务的合法权利方面至关重要，因为它们会影响其可及性和财政支持情况。

**结论:**

该分析为政策制定者提供了见解，以评估确保健康相关应享有权利的不同方法，从而对卫生筹资和评估成员国在全面普及综合医疗服务方面所取得的进展产生影响。这项分析还阐明了不同的健康权法规对减少健康差异有何不同影响。

## Резюме

**Цель:**

Изучить влияние различных формулировок права на здоровье в национальных конституциях, законодательстве и судебной практике на сферу правовой защиты здоровья.

**Методы:**

Для изучения юридического признания права на здоровье был проведен трехуровневый поиск. Во-первых, был проведен поиск по базам данных, содержащим конституционные тексты. Во-вторых, был проведен тематический анализ конституционных текстов, содержащих прямое конституционное признание прав на здоровье, с использованием программного пакета NVivo для кодирования. Для 54 государств-членов Всемирной организации здравоохранения (ВОЗ), в которых отсутствуют четкие конституционные положения, были изучены законодательные акты, судебные конструкции и случаи, когда оба метода способствовали признанию прав на здоровье. В завершение было подтверждено наличие юридической практики, определяющей право на здоровье на основе сочетания внутреннего законодательства и международных норм в области прав человека, включенных непосредственно в текст.

**Результаты:**

Было выявлено 140 государств-членов ВОЗ, в которых право на здоровье закреплено в тексте конституции. Результаты проведенного нами анализа свидетельствуют о наличии значительных различий в сфере правовой защиты здоровья, включая широту прав и возможность реализации этих прав через правовую систему. Кроме того, важно отметить решающее значение конституционного признания, законодательных мер и судебных толкований в формировании правовых прав на медицинские услуги, влияющих на их доступность и финансовую поддержку.

**Вывод:**

Результаты анализа позволяют ответственным лицам оценить различные подходы к выплатам, связанным со здоровьем, с последствиями для финансирования здравоохранения и оценки шагов государств-членов по обеспечению всеобщего доступа к комплексному медицинскому обслуживанию. Результаты анализа также свидетельствуют о том, что различные формулировки права на здоровье оказывают разное влияние на сокращение неравенства в сфере здравоохранения.

## Introduction

In 1946, the newly drafted Constitution of the World Health Organization (WHO) recognized the fundamental right to enjoy the highest attainable standard of health without distinction of race, religion, political belief, economic or social condition.^1^ Since then, human rights have grown more central to global health discussions. They are widely seen as vital tools for combating health disparities and fostering fairness in health care.^2^ Substantial evidence suggests that adopting the right to health through national constitutions, legislation and/or judicial interpretation, can positively influence population access to health goods, facilities, technologies and services; and curb discrimination and abuse within health systems.^3^^–^^6^ However, while exhortations to ratify international treaties have grown common, the impacts of enshrining legal entitlements have varied widely. Past studies have shown that the legal recognition of the right to health, while critical, does not guarantee universal access to comprehensive health care.^7^^,^^8^

We recognize that gaps in compliance and regulation can hinder the full exercise of health rights. However, our study focuses on the different legal grounds and definitions of these rights, and the responsibilities of governments. These rights are defined by laws or court decisions and can shape the extent of legal health protection. We consider these rights formulations as separate factors when examining policies and government funding for health care. To evaluate the influence of right to health on health-care financing and the goal of universal care access, diverse legal formulations and sources need to be considered. Comparisons of health-care spending that only account for the existence of a constitutional right to health might overlook significant nuances and lead to inaccurate conclusions. Hence, this article introduces an analytical method designed to clarify how health rights recognition, coverage levels and health financing are interconnected

Based on quantitative and qualitative research performed with the WHO Council on the Economics of Health for All,^9^ this article proposes an analytical framework that examines the mechanism for legal recognition, as well as elements included in the textual formulation of the right to health. Our framework uses three stages of analysis. The first stage interprets the legal recognition of the right to health (constitutional, legislated, judicial or a combination). If there is some constitutional recognition, the second step explores alternative framings of a constitutional right, that is: (i) explicit references to basic services; (ii) rights set out as programmatic aspirations or directive principles; (iii) an explicit reference to other laws or regulations to enact the right to health; (iv) explicit reference to free health care; (v) explicit obligation to protect the health of people; (vi) explicit reference to public health; and (vii) mechanisms of resources and financing. The third stage examines the availability of alternative or additional legislative and judicial mechanisms for the construction of health rights.

Our method enhances research and policy development by examining more than just whether a country constitutionally recognizes the right to health. By disaggregating forms of recognition, the constitutional framing and the content of alternative legal or judicial adoption of health rights, the framework can assist in understanding the legal protection's breadth, enforceability and implications for health financing. By looking into these elements, our method allows us to investigate the various approaches taken by legal systems in managing the funding of health-care services.

## Methods

We analysed the constitutions of all 194 WHO Member States. To do this analysis, we searched for the most recent constitution in the databases of Constitute Project, Venice Commission, WIPO Lex, International Labour Organization Library, and Pacific Islands Legal Information Institute (Paclii). To obtain the most up-to-date set of texts for analysis, a single author extracted the most recent versions of constitutional texts from the aforementioned databases. We extracted and examined only the latest versions of constitutional text, including all amendments and reforms to the constitutional texts as of December 2023.

The databases searched provide constitutional texts in English and we therefore collected all documents in English. We consolidated segments of the text related to the right to health into a collection in NVivo, version 12 (Lumivero, Denver, United States of America), where we systematically reviewed the text for terms related to health and what kind of entitlements to health care or public health were included in the constitutional text. Using NVivo, we coded themes attributed to scope or enforceability of constitutional rights through an inductive and iterative process, examining common framings and variations in provisions associated with health care and public health. 

We created coding themes related to scope based on contextualized, bottom-up iterative reading and interpretation of the data. We then organized coding outcomes into a data matrix featuring: (i) WHO Member State; (ii) citation of the constitutional source; and (iii) an excerpt of the relevant constitutional text. [Fig F1]

**Fig. 1 F1:**
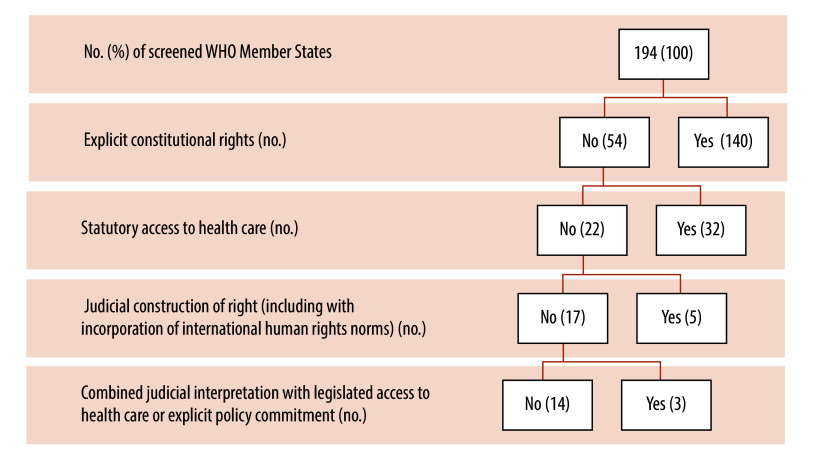
Flowchart of identifying WHO Member States with constitutionalized right to health

WHO: World Health Organization.

Note: Of the three Member States we identified as having combined judicial interpretation with legislated access to health care or explicit policy commitment, two states had explicit policy commitment and one state had combined legislated judicial recognition.

For WHO Member States lacking any explicit constitutional right to health, a second level of analysis was initiated to explore whether there was a statutory right to health or a judicially constructed constitutional right, including those based on incorporation of international law directly into constitutional texts. We examined databases of domestic legislative bodies and other government websites for statutory rights. As no reliable indexed official directory exists for governments, except for small Island States in the Pacific (Paclii database), we manually searched for statutory rights for each jurisdiction we studied. We also searched Google to identify government websites where legitimate versions of judicial opinions are published, interpreting statutory rights and academic literature necessary for understanding domestic jurisprudence; focusing on affirmative entitlements to health care. Text in languages other than English were unofficially translated by the authors or by using Google Translate. We have provided access to our collection of detailed legal sources in a public repository.^10^

For Member States with no statutory right to health identified, we conducted a third level of analysis. We searched the HeinOnline database and Google Scholar for English documents to confirm evidence of jurisprudence constructing a right to health based on a combination of domestic law and international human rights norms incorporated directly into the text. Only texts from Argentina, Germany and Mauritania were in official languages other than English, requiring support of Google Translate. In keeping with accepted methods of comparative legal research, a single reported case of judicial enforcement was sufficient to establish the existence of jurisprudence, which was determined by entering the following search terms: (i) right to health; (ii) court; and (iii)  the name of the jurisdiction. 

For the final stage of analysis, we fed the data set generated by our three-level search into a data matrix for each WHO Member State, including: (i) the legal basis for the right to health; (ii) the category of incorporation (constitutional, statutory, judicially constructed, or a combination); (iii) the year of the last revision or amendment of the legal source (where applicable); and (iv) an extract of the constitutional text containing the reference to the right to health. 

## Results

Our analysis reveals a multifaceted distribution of recognized rights to health among WHO Member States. Initial analysis at the constitutional level uncovered explicit provisions related to at least one aspect of the coding theme in 140 constitutions. Consequently, these 140 Member States were identified as having a constitutional right to health (Box 1). Among Member States lacking a constitutional provision, 32 Member States had a recognized health-care entitlement through legislation, while five had obtained recognition through judicial means. We could not find data related to the right to health either in constitutional law, legislation or judicial cases in 14 of the 194 Member States (Fig. 1).

Box 1 **WHO Member States with a constitutionalized right to health**

Afghanistan, Albania, Algeria, Andorra, Angola, Armenia, Azerbaijan, Bahrain, Bangladesh, Belarus, Belgium, Belize, Benin, Bhutan, Bolivia (Plurinational State of), Bosnia and Herzegovina, Brazil, Bulgaria, Burkina Faso, Burundi, Cabo Verde, Cambodia, Central African Republic, Chile, Colombia, Comoros, Congo, Côte d'Ivoire, Croatia, Cuba, Czechia, Democratic People's Republic of Korea, Democratic Republic of the Congo, Dominican Republic, Ecuador, Egypt, El Salvador, Equatorial Guinea, Eritrea, Estonia, Eswatini, Ethiopia, Fiji, Finland, Gabon, Gambia, Georgia, Greece, Guatemala, Guinea, Guinea-Bissau, Guyana, Haiti, Honduras, Hungary, Indonesia, Iran (Islamic Republic of), Iraq, Italy, Japan, Kazakhstan, Kenya, Kuwait, Kyrgyzstan, Lao People’s Democratic Republic, Latvia, Lesotho, Liberia, Libya, Lithuania, Luxembourg, Madagascar, Malawi, Maldives, Mali, Marshall Islands, Mexico, Micronesia (Federated States of), Mongolia, Montenegro, Morocco, Mozambique, Myanmar, Namibia, Nepal, Netherlands (Kingdom of the), Nicaragua, Niger, Nigeria, Niue, North Macedonia, Oman, Pakistan, Palau, Panama, Papua New Guinea, Paraguay, Peru, Philippines, Poland, Portugal, Qatar, Republic of Korea, Republic of Moldova, Romania, Russian Federation, Rwanda, Sao Tome and Principe, Saudi Arabia, Senegal, Serbia, Seychelles, Sierra Leone, Slovakia, Slovenia, Somalia, South Africa, South Sudan, Spain, Sri Lanka, Sudan, Suriname, Switzerland, Syrian Arab Republic, Tajikistan, Thailand, Timor-Leste, Togo, Tunisia, Türkiye, Turkmenistan, Uganda, Ukraine, United Republic of Tanzania, Uruguay, Uzbekistan, Venezuela (Bolivarian Republic of), Viet Nam, Yemen and Zimbabwe.

WHO: World Health Organization.

Further examination identified three Member States that did not fit into the aforementioned screening categories. Notably, in one case, a blend of judicial interpretation and legislative measures culminated in the establishment of a political recognition of the existence of a right to health. In contrast, in two other cases, governmental policies explicitly articulated rights language, thereby implicitly adopting entitlements to health care.

By analysing different constitutions mentioning the right to health, we identified common themes among them; that is, free health care, emergency health care, basic health-care services and health protection. We selected two coding themes related to legal enforceability: (i) provided by law; and (ii) directive principles, as contrasted with fundamental rights in many constitutional texts.

### Variations in provisions

When Member States ratify international treaties or independently move to enshrine entitlements to the right to health, different rights formulations produce differing scopes of legal protection and entitlement. These in turn influence how health systems are financed and organized.

Across Member States, key variations in constitutional provisions include an explicit reference to free health care; rights set out as programmatic aspirations or directive principles; or as justiciable fundamental rights. We also reviewed if basic, emergency or comprehensive care was stipulated in the text, and by what mechanisms financing is embedded in the text of the Constitution. These variations are described in detail in Box 2.

Box 2 **Key variations in constitutional provisions regarding the right to health**

**Explicit reference to basic services**

A constitutional right to health may be expressed as a right to basic or essential health-care services (for example, Armenia; Constitution of the Republic of Armenia, Art. 85) or emergency health services. Essential health services are not defined in constitutional texts and may differ from provisions in soft law instruments under international law. Constitutional provisions for emergency health services do not guarantee that after receiving care, patients will not be charged.

**Rights set out as programmatic aspirations or directive principles**

A right to health may be established under a set of social objectives or directive principles to be interpreted as collective aspirations rather than fundamental^5^^,^^6^ rights with full individual enforcement and protections (for example, Belarus; Constitution of the Republic of Belarus of 1994, Art. 45; and Bhutan; Constitution of Bhutan, Art. 21). These do not bind the state to making claims enforceable nor to progressive realization of rights.

**Explicit reference to other laws or regulations to enact the right to health**

In 62 constitutions, implementation of state obligations or policies is contingent upon subsequent legislation or regulation. This pattern means that in certain cases the constitutional text does not take effect if there is no further legislative action or regulation.

**Explicit reference to free health care**

We found explicit provision of free health care in 44 constitutional texts; out-of-pocket payments are permissible even when there is an explicit right to health care. Some provisions stipulate free health care only for basic services or for people in extreme economic need (for example, Tajikistan; Constitution of the Republic of Tajikistan, Art. 38).

**Explicit obligation to protect the health of people**

In certain instances, constitutional provisions are articulated in a manner that suggests the obligation of protection, which can be construed as aiming to prevent harm from various sources, such as those seen in sanitary laws or labour health standards. We identified this language in 33 constitutions.

**Explicit reference to public health**

This category comprises constitutions that incorporate coding related to state policies and responsibilities concerning population health, which go beyond mere treatments or medical care. They encompass initiatives such as disease prevention, epidemiological surveillance, and measures for health promotion or risk reduction. We identified this framing in 71 constitutions.

**Resource allocation and financing**

The sources of financing or the rules of health-care expenditure are rarely explicitly set out in a constitution. Exceptions to this rule include Member States such as Brazil (Constitution of the Federative Republic of Brazil, Art. 196); and Colombia (Political Constitution of Colombia, Art. 49), both of which detail regulation of health-care financing. Ecuador (Constitution of the Republic of Ecuador, Transitional Provisions, Art. 18); and Egypt (Constitution of the Arab Republic of Egypt, Art. 18) earmarked 4% and 3% of their GDP towards health care, respectively.

GDP: gross domestic product.

Moreover, the framing of the right itself affects resource allocation and health governance. For example, in Albania there is a right to health insurance whereas in Brazil, there is a universal right to health care as a judicially enforceable entitlement. Thus, in Albania, type of insurance defines coverage and the scope of the right. By contrast, in Brazil, there is universal access (regardless of immigration status) to the publicly funded health system *Sistema Único de Saúde*.

### Alternative pathways

Even if there is no constitutional provision regarding the right to health, the right may be established by incorporating international human rights norms in legislation or through judicial construction in the constitutional text, (for example, Argentina; Political Constitution of the Republic of Argentina, Articles 14(bis), 41, and 75). Judicial construction of health-related rights occurs through iterative interpretation of the constitutional text, a process informed to differing degrees by international human rights law. Courts may derive a positive right to health based on one or more other rights; that is, (i) life; (ii) self-determination; (iii) equality or non-discrimination; and (iv) consumer protections. Courts can also interpret directive principles as fundamental rights.^11^^,^^12^ For example, in Costa Rica and India, apex courts have constructed a right to health, including health care, as part of the right to life with dignity. 

In theory, these judicial constructions have the same weight as an explicit constitutional provision on the right to health. However, they can also be more narrowly circumscribed to certain types of care. For instance, in Uganda, there is a judicially constructed right to maternal health care but not other types of care.^13^^,^^14^ Even when some aspect of the right to health is set out in the constitution or a statute, it is frequently translated into concrete entitlements through judicial interpretation. In turn, judicial construction often calls for legislation or public policies to make changes in a health system.^13^ Sometimes courts create affirmative entitlements to forms of health care by limiting the authority of the legislature; that is, with respect to criminalizing abortion.^15^^,^^16^

In short, consistent with other research on social rights, the degree to which a right to health implies universal or differentiated entitlements, and the degree to which it is effective at influencing public policies and programmes depends upon constitutional parameters, judicial construction and legislative action.^17^

## Discussion

Assessing the realization of health rights and related public spending by merely counting Member States with constitutional health rights can yield misleading conclusions. For example, Argentina has no explicit constitutional right to health per se. However, a right to health with substantial policy and spending implications has been judicially enforced through other constitutional rights and the incorporation of international norms to which Argentina is a party.^7^ By contrast, other studies have found that the mere existence of an explicit right to health in many constitutions does not necessarily lead to material advances in health-care financing; or to access to health-care related goods, facilities and services.^18^

Different articulations of rights carry different implications for universality and comprehensiveness, as well as progressivity. For example, a right to health based on social insurance through employer and employee contributions is inherently influenced by structural factors such as the percentage of self-employed workers engaged in the informal economy. Likewise, a universal right circumscribed to basic care has implications for health governance, priority-setting and financing of the health system, as unfunded services may then out of necessity be provided by costly private health-care providers.

Constitutions are frequently amended; and legislation and judicial interpretations of health rights continually evolve based upon both normative and empirical shifts. That is, the emergence of a new disease or the advent of a life-saving technology can change the interpretation of the scope of the constitutional right, as can social norms around issues such as abortion or gender-affirming care.^6^^,^^19^^–^^21^

In contrast, our three-stage analysis allows for deeper considerations of context than a simple tally. For example, courts deploy interpretations of other constitutional norms, such as the right to life, consumer protections, and equality and non-discrimination to enforce regulations of public and private actors regarding the scope and content of health entitlements in specific contexts. Thus, judicial interpretation, even in the absence of an explicit right to health, is critical for effective deployment of health entitlements by the general population as well as disadvantaged and marginalized groups.^18^

Our emphasis on the role of courts in rights realization complements findings from other research, showing that judicial capacity to ensure compliance with entitlements to health care varies, including when care is provided by private and not state entities.^11^^,^^22^ Enforcement of health-related rights depends not just upon constitutional or statutory texts but also awareness of rights violations (i.e. legal consciousness), the need for and availability of counsel, rules regarding standing, costs of filing a claim and state compliance with judicial orders.^23^^–^^28^ We also note that entitlements to health care may be enforced by courts in ways that either undermine systemic equity and systematic priority-setting, or act to enhance the fairness and deliberative quality of that priority-setting process.^7^

One area of future investigation is to determine how the framing of the right to health may contribute to shifting social norms and the restructuring of health systems as social institutions. For example, in countries such as Brazil that have adopted a constitutional right to universal and comprehensive health care, issues in the health system are no longer merely technical questions about quality of care, but have progressed to more serious issues of fundamental human rights and dignity.^28^^,^^29^ Comparative research designed for policy-makers that connects these epistemic and structural effects on conceiving of health as a common good with specific public policies and health outcomes across populations could be useful in mobilizing greater pooled public resources for financing health, as opposed to reliance on private insurance markets.

This study has some limitations. First, we primarily used constitutional directories available in the English language. Second, there is no centralized or officially indexed research database for all legislation and judicial decisions concerning the right to health. Some sources may not be published by governments or available online; and if so, may have not been filed in the legal research inventories we consulted. 

In conclusion, to effectively assess the right to health's influence on health-care financing and social outcomes, policy and public health researchers can employ a more detailed analytical framework. We found that our analytical method facilitates a more thorough understanding of the breadth and consistency of legal protections in Member States. 

Linking the findings of this study with the report of the WHO Council on the Economics of Health for All^30^ suggests a broader research agenda that examines the interactions among the enshrinement of health rights; prevailing social norms around health equity and solidarity; and the institutionalized economic order in some countries such as intellectual property regimes and financial regulation. Although consideration of the social determinants of health and socio-legal context is necessary for full evaluations, these different framings will have substantial implications for WHO Member States in terms of health system financing, governance and priority-setting processes.
